# The relevance of outcome expectations in group hypnosis for stress reduction: a secondary analysis of a multicenter randomized controlled trial

**DOI:** 10.3389/fpsyg.2024.1363037

**Published:** 2024-04-19

**Authors:** Julia Siewert, Michael Teut, Benno Brinkhaus, Silvia Fisch, Sonja Kummer

**Affiliations:** ^1^Institute of Social Medicine, Epidemiology and Health Economics, Charité – Universitätsmedizin Berlin, Berlin, Germany; ^2^Psychotherapie-Praxis Kupferstraße, Coesfeld, Germany

**Keywords:** hypnosis, hypnotherapy, stress, stress reduction, outcome expectation, randomized controlled trial

## Abstract

**Background:**

There is evidence that patients’ positive outcome expectations prior to study interventions are associated with better treatment outcomes. Nevertheless, to date, only few studies have investigated whether individual outcome expectations affect treatment outcomes in hypnosis.

**Objective:**

To examine whether outcome expectations to hypnosis prior to starting treatment were able to predict perceived stress, as measured on a visual analog scale (VAS), after 5 weeks.

**Methods:**

We performed a secondary data analysis of a multicenter randomized controlled trial of intervention group participants only. Study participants with stress symptoms were randomized to 5 weekly sessions of a group hypnosis program for stress reduction and improved stress coping, plus 5 hypnosis audio recordings for further individual practice at home, as well as an educational booklet on coping with stress. Perceived stress for the following week was measured at baseline and after 5 weeks using a visual analog scale (0–100 mm; VAS). Hypnosis outcome expectations were assessed at baseline only with the Expectations for Treatment Scale (ETS). Unadjusted and adjusted linear regressions were performed to examine the association between baseline expectations and perceived stress at 5 weeks.

**Results:**

Data from 47 participants (M = 45.02, SD = 13.40 years; 85.1% female) were analyzed. Unadjusted (B = 0.326, *t* = 0.239, *p* = 0.812, *R*^2^ = 0.001) and adjusted (B = 0.639, *t* = 0.470, *p* = 0.641, *R*^2^ = 0.168) linear regressions found that outcome expectations to hypnosis were not associated with a change in perceived stress between baseline and after 5 weeks in the intervention group.

**Conclusion:**

Our findings suggest that the beneficial effect of group hypnosis in distressed participants were not associated with outcome expectations. Other mechanisms of action may be more important for the effect of hypnosis, which should be explored in future research.

**Clinical trial registration**: ClinicalTrials.gov, identifier NCT03525093.

## Background

The European Agreement on Work-Related Stress defines stress as a state characterized by physical, psychological, or social complaints or dysfunctions resulting from individuals feeling unable to meet the demands or expectations placed upon them (Broughton, 2004). This definition underscores the global recognition of health-related problems associated with stress (Fisch et al., 2020a; Gnall et al., 2023; Mazure et al., 2023; Popescu et al., 2023; Sara et al., 2023; Walther and Wirtz, 2023). In Germany, a survey of 1,200 adults found that 61% reported experiencing stress either frequently or occasionally (Wohlers and Hombrecher, 2016).

Hypnosis is a state of focused attention and heightened suggestibility that can be induced by a trained professional. It has been used as a therapeutic tool for a variety of clinical purposes, including stress reduction. A 2017 systematic review examined the effects of hypnosis in patients with perceived stress. While six of the nine included studies reported significant positive effects of hypnosis on stress reduction, all of the included studies had a high risk of bias and used exploratory designs (Fisch et al., 2017). Since the publication of the aforementioned review, our research group has conducted a multicenter randomized controlled trial offering group hypnosis for stress reduction and improved stress coping, which showed a reduction in perceived stress in the hypnosis group compared to the control group at both 5 and 12 weeks (Fisch et al., 2020a). Other studies have also shown that hypnosis leads to a lower perception of stress (Payrau et al., 2017; Olendzki et al., 2020; Slonena and Elkins, 2021; Vahdat et al., 2022).

Although scientific evidence from other fields suggests that expectations are able to positively influence treatment outcomes for a range of medical conditions and procedures (Mondloch et al., 2001; Constantino et al., 2011; Auer et al., 2016), little research has examined whether expectations to hypnosis might be able to predict treatment outcomes.

Patients’ expectations may encompass their beliefs about the efficacy of hypnosis and their anticipated outcomes from the treatment in question. To date, few studies have attempted to discern whether individuals with high expectations of positive outcomes to hypnosis will experience more significant benefits compared to those with low expectations (Sliwinski and Elkins, 2017; De Pascalis et al., 2021; Egli et al., 2022). This debate is imperative because it raises questions about the role of psychological factors in the therapeutic process and the validity of hypnosis as a treatment modality in its own right. A perspective that has not been very well researched suggests that patients with different expectations to hypnosis may experience a different therapeutic effect, whereby their belief in the efficacy of the treatment may influence their response to it (Frisaldi et al., 2015; Koban et al., 2017). In this respect, participants’ expectations may confound the interpretation of study results, making it challenging to isolate the specific effects of hypnosis itself. As indicated by other studies, expectations also contribute to placebo and nocebo effects (Petrovic et al., 2005; Wager et al., 2007; Tracey, 2010). Consequently, they could also influence the effectiveness of hypnotic interventions (Kirsch, 1985). Moreover, it is assumed that hypnosis in clinical practice can induce altered states of consciousness independently of initial expectations and produce therapeutic benefits through suggestion and relaxation techniques.

We performed a secondary data analysis to examine whether treatment expectations to a group hypnosis program for stress reduction and improved stress coping would be able to predict perceived stress in the previous week, as measured on a visual analog scale, after 5 weeks.

## Methods

### Study design

This study comprised a secondary analysis of the two-armed randomized, controlled, open, multicenter HypnoStress trial (Trial Registration No. NCT03525093; Ethical Approval No. EA1/067/18). Details of the original study have been published elsewhere (Fisch et al., 2020a). This paper reports findings from a secondary data analysis only and required no additional ethical approval.

### Participants and recruitment

Individuals were considered eligible for participation in the original trial if they were aged between 18 to 70 years, reported a subjective stress level of 40 mm or higher on a visual analog scale (VAS) for the preceding week (measured on a scale of 0 to 100 mm), reported a perceived increase in stress lasting for at least 3 months, maintained overall good health, and provided written informed consent. Conversely, individuals were excluded if they were currently participating or planning to participate in another psychological stress reduction program within the next 12 weeks, were currently undergoing psychotherapy, had a moderate or severe acute or chronic medical condition, or had an acute or chronic mental health problem. Recruitment for the study was conducted via newspaper ads in Berlin and Coesfeld, the Charité Outpatient Department for Integrative Medicine’s website and newsletter, the psychotherapeutic clinic in Coesfeld, the Studienhospital Münster’s Newsletter, and flyers at the MEDIAN Zentrum Bad Pyrmont. Potential participants underwent a preliminary consultation with a psychologist or study physician, where they were informed about the study.

### Randomization

A detailed summary of the randomization and intervention content is provided in the original article (Fisch et al., 2020a). Briefly, patient enrollment was conducted under the supervision of study physicians and study psychologists. Following informed consent, enrollment and baseline assessments, participants were randomized to either the intervention or control group, using a 1:1 allocation ratio via a central telephone randomization line by an independent study nurse. The randomization was stratified by study center and in blocks of 20 participants (to take into account the group size of 10 people). SAS (Version 9.4) was used to generate the random allocation sequence.

### Study intervention

Both the intervention and control group received a written educational booklet on behavioral stress management provided by a German health insurance company (Wagner-Link, 2017). The booklet contained sections on “recognizing stress,” “managing stress,” and “preventing stress.” The “recognizing stress” section outlined the physiological underpinnings of a natural stress response, detailing various facets of stress reactions, including cognitive, emotional, vegetative, and muscular aspects. It also aimed to sensitize readers to identify individual stressors. In the “managing stress” section, common stress management strategies such as problem-solving, time management, various relaxation techniques, sports, and recognizing and modifying unfavorable attitudes were introduced and briefly discussed. The third section, “preventing stress,” introduced the salutogenesis model and provided insights into the structure and promotion of resilience factors, with a particular emphasis on maintaining social connections. Additionally, this section outlined short-term stress management strategies and offers a suggested training protocol (Wagner-Link, 2017; Fisch et al., 2020b).

In addition to this, the intervention group received a hypnosis group program, which was previously designed, refined and tested in a feasibility study (Fisch et al., 2020b). The primary objectives of the hypnosis group program were to induce relaxation, assist participants in recognizing, activating, and experiencing resources for coping with stressful situations, foster the development and refinement of stress-coping skills, and impart mental training and anchoring techniques. The program was delivered by certified hypnotherapists (two psychotherapists and one family physician) and consisted of five standardized sessions of health education, hypnotic inductions, and therapeutic discussions. Hypnosis sessions were conducted weekly with groups of 8 to 12 participants and lasted 120 min. Additionally, at the end of each session, participants were provided with pre-recorded audio recordings (available as either CDs or downloadable MP3 files) of the hypnosis exercises so that they could self-practice at their convenience and discretion. Control group participants were offered free participation in the hypnosis group program following study completion.

### Outcome measures

Relevant outcomes for this secondary data analysis were:

*Stress*: perceived stress level in the previous week was measured on a visual analog scale (VAS; 0–100 mm: 0 = no stress, 100 = maximum stress) after 5 weeks.
*Outcome expectations*: expectations to hypnosis treatment were measured using a modified version of the Expectations for Treatment scale (ETS) (Barth et al., 2019). Participants were asked to indicate their expectations to hypnosis on a Likert scale ranging from 1 (low expectations) to 4 (high expectations): *“I expect that hypnosis will help me deal with stress better,” “I expect stress levels to disappear as a result of hypnosis,” “I expect my energy to improve as a result of hypnosis,” “I expect my physical performance to improve as a result of hypnosis,” “I expect that after the hypnosis stress levels will improve significantly.”* Items were summed to create a total score, with a minimum score of 4 indicating low expectations and a maximum score of 20 indicating high expectations.
*Demographic* *variables*: self-reported data on age, gender, education, employment status, health parameters and stress factors were obtained at baseline.

### Statistical analysis

The ETS was collapsed into a dichotomous variable using the median value (*MD* = 13.00) as the cut-off to group individuals into high (if the median score was above *MD* = 14.00) and low (if the median score was below *MD* = 13.00) expectations in order to determine and display baseline group differences regarding expectations to hypnosis only. Baseline group differences for sociodemographic, health and stress-related characteristics were analyzed using t-tests for continuous data and chi-square tests or Fisher’s exact test for small cell counts for categorical data, and summarized using means, SDs, or percentages.

Unadjusted linear regressions were then calculated to examine whether expectations to hypnosis (for this the ETS sum score was used) in the intervention group would be able to predict change in perceived stress between baseline and after 5 weeks as measured on a VAS. Linear regressions were subsequently adjusted for any potential confounders (baseline stress, study center, age, and sex). To determine whether expectations to hypnosis (for this the ETS sum score was used) in the intervention group would be able to predict change in perceived stress between baseline and after 5 weeks, we performed a sensitivity analysis using Spearman’s rho correlation to examine whether this non-parametric alternative would yield similar results as the linear regression. All results were considered exploratory. Analyses were conducted using the IBM Statistical Package for Social Sciences (SPSS), Version 28.

### Sensitivity analysis

Two sensitivity analyses were conducted to test the robustness of our results. For the first we performed a non-parametric correlation analysis using Spearman’s rank-order correlation to determine expectations to hypnosis and change in perceived stress between baseline and after 5 weeks. For the second we performed unadjusted and adjusted regression analyses using the change in Cohen’s Perceived Stress Scale (CPSS) (Cohen et al., 1983) score as an outcome.

## Results

Detailed sociodemographic characteristics of the sample are outlined in the original study article (Fisch et al., 2020a). Table 1 shows the comparison of sociodemographic characteristics between those with high and low expectations to hypnosis in the intervention group. We observed no relevant differences at baseline in individuals with high and low expectations.

**Table 1:** Comparison of sociodemographic characteristics between those with low versus high expectations in the intervention group (baseline).

	Intervention group	
	Low expectations *N* = 23	High expectations *N* = 24
	Mean ± SD / *n* (%)	Mean ± SD / *n* (%)
VAS Baseline Stress	72.78 (11.14)	74.71 (9.18)
Age [years]	43.26 (13.57)	46.71 (13.30)
Sex [Female]	21 (91.3)	19 (79.2)
Education		
Abitur (German university entrance qualification)	17 (73.9)	19 (79.2)
Employment		
Employed [yes]	20 (87.0)	21 (87.5)
Household		
Single person household	5 (21.7)	6 (25.0)
Two-person household	6 (26.1)	10 (41.7)
Multiple person household	12 (52.2)	8 (33.3)
Health parameters		
Smoking [yes]	4 (17.4)	3 (12.5)
Alcohol [yes]	19 (82.6)	18 (75.0)
Physical activity [yes]	22 (95.7)	22 (91.7)
Physical activity frequency [1–2 times per week]	11 (50.0)	12 (54.5)
*Stress factors (multiple responses possible)*		
Professional factors		
Job/School/University	16 (69.6)	17 (70.8)
Exam preparation	6 (26.1)	4 (16.7)
High demands on oneself	17 (73.9)	18 (75.0)
Conflicts with colleagues /superiors	5 (21.7)	1 (4.2)
Time pressures, high density of appointments	17 (73.9)	15 (62.5)
Private factors		
Private conflicts	6 (26.1)	12 (50.0)
Parenting	4 (17.4)	5 (20.8)
Disease (loved one)	9 (39.1)	4 (16.7)
Caring for a relative	3 (13.0)	2 (8.3)
Money worries	3 (13.0)	2 (8.3)
Household	5 (21.7)	4 (16.7)
Preparation of special events/festivities	4 (17.4)	1 (4.2)
Adversities of everday life/daily hassles		
Organizing everyday activities	4 (17.4)	9 (37.5)
Public transport	4 (17.4)	3 (12.5)
Doctor visits	3 (13.0)	3 (12.5)
Waiting	1 (4.3)	0 (0.0)
Being disturbed/interrupted	6 (26.1)	10 (41.7)
Other	5 (21.7)	5 (20.8)

Ns may vary in each cell due to missing data.

**Figure 1:**
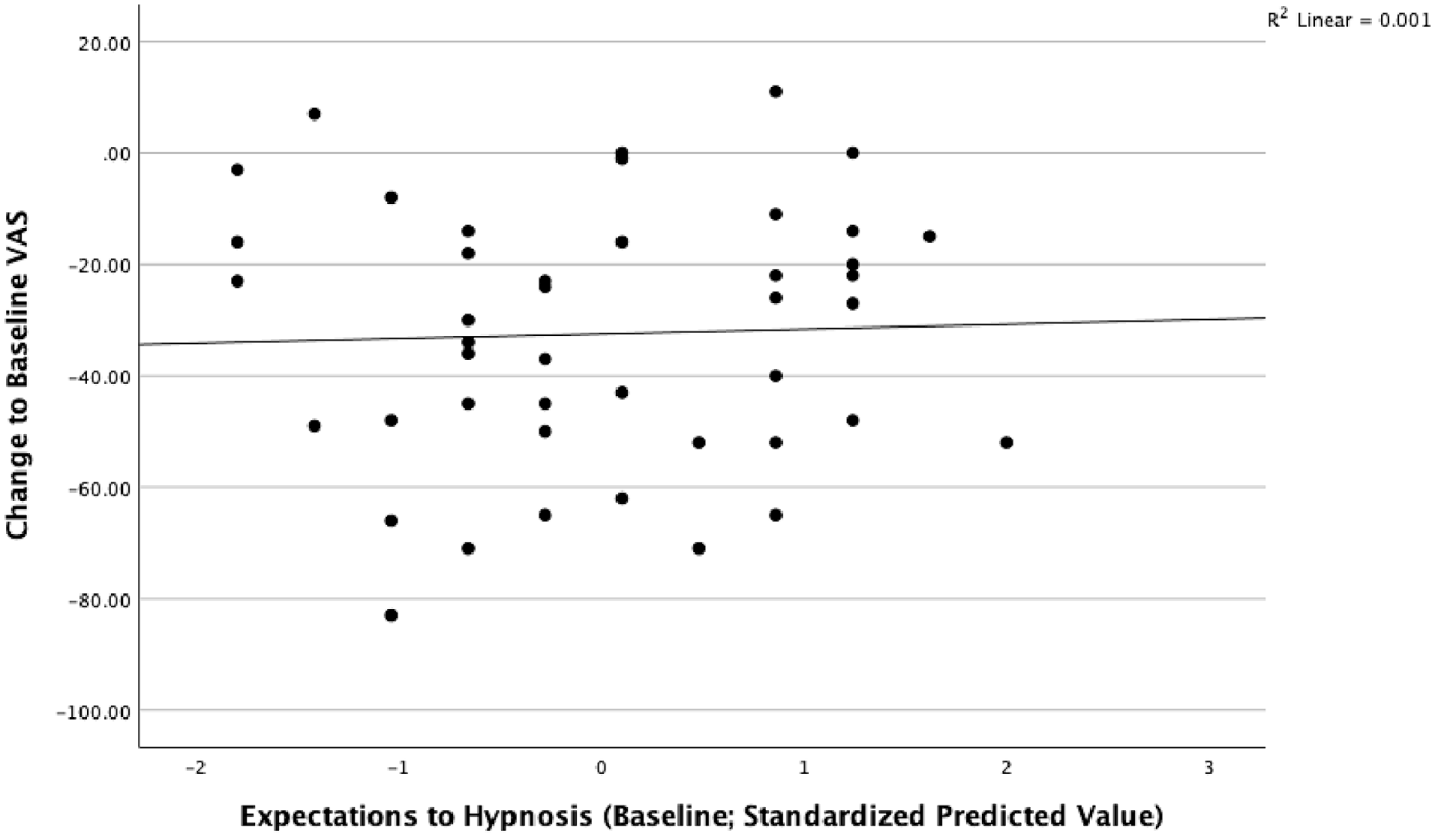
Expectations to hypnosis and perceived stress after 5 weeks in the intervention group.

Unadjusted linear regressions showed that expectations to hypnosis were not associated with a change in perceived stress between baseline and after 5 weeks (*B* = 0.326, *t* = 0.239, *p* = 0.812, *R*^2^ = 0.001) (Figure 1).

**Figure 2:**
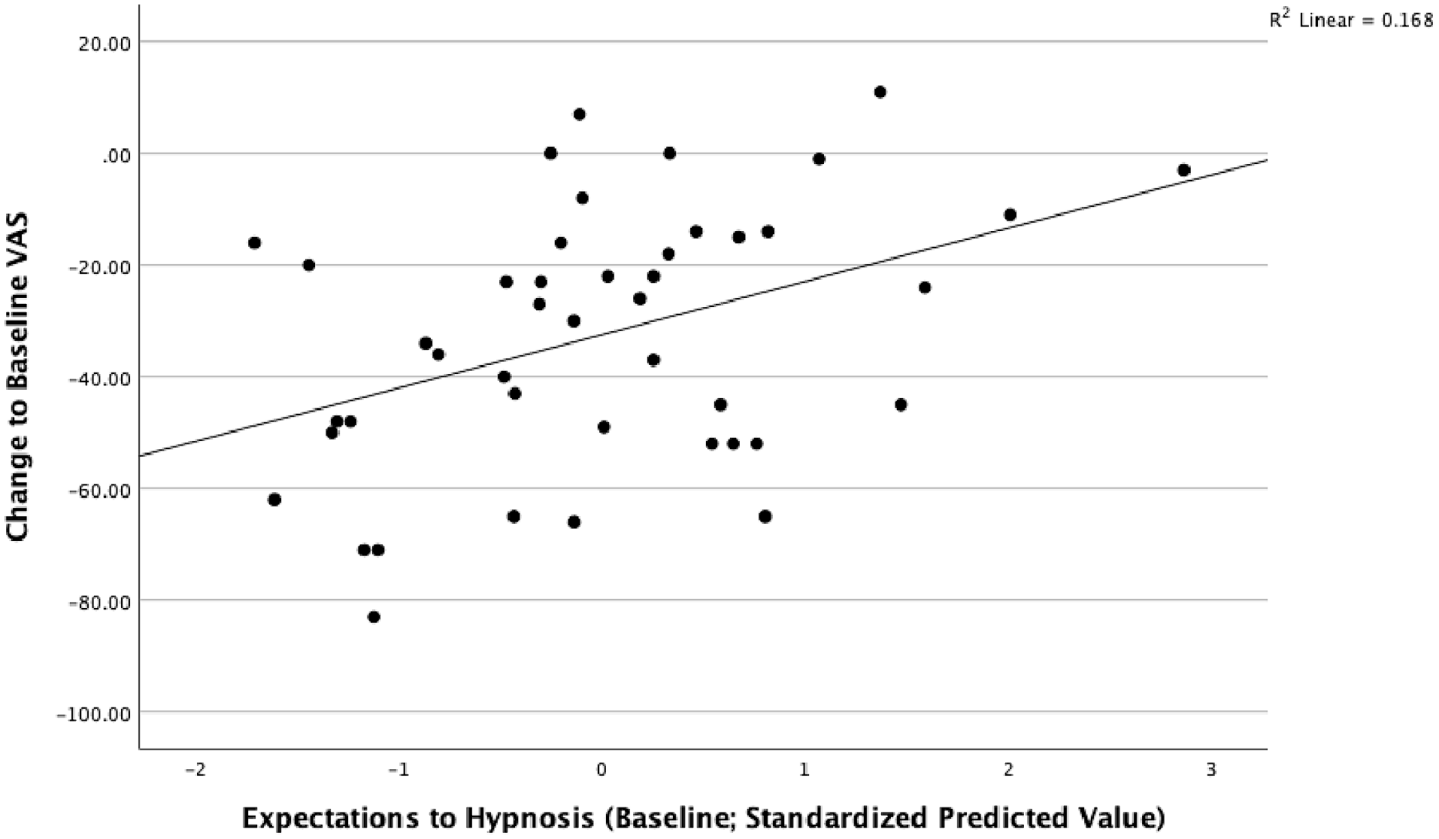
Expectations to hypnosis and perceived stress after 5 weeks in the intervention group; adjusted for respective baseline value, study center, age and sex.

Similarly, adjusted linear regressions showed that expectations to hypnosis were not associated with a change in perceived stress between baseline and after 5 weeks (B = 0.639, *t* = 0.470, *p* = 0.641, *R*^2^ = 0.168) (Figure 2).

### Sensitivity analysis

Sensitivity analysis using Spearman’s rank-order correlation indicated no relevant relationship between expectations to hypnosis and change in perceived stress between baseline and after 5 weeks (r_s_ = 0.06, *p* = 0.703).

Similar to our other findings unadjusted (B = −0.350, *t* = −0.870, *p* = 0.389, *R*^2^ = 0.018) and adjusted (B = −0.118, *t* = −0.341, *p* = 0.735, *R*^2^ = 0.400) linear regressions found that outcome expectations to hypnosis were not associated with a change in perceived stress between baseline and after 5 weeks in the intervention group on the CPSS.

## Discussion

Contrary to previous research that has shown that expectations predict treatment outcomes (e.g., Auer et al., 2016), our findings showed no association between participants’ expectations and perceived stress after 5 weeks. Consequently, other contextual factors, such as hypnotic relaxation, active resource activation, and reframing techniques and group interactions may have played a greater role than expectations in determining treatment outcomes.

Our results showed that overall participants had relatively high expectations at baseline (M = 13.74, SD = 2.72) before being randomized to and participating in the group hypnosis program. Therefore, it could be suggested that future studies should include individuals with more diverse expectations in order to determine how these may be associated with varying treatment outcomes. For example, research on recovery expectations in patients with back pain (Kamper et al., 2015) has shown that the level of expectations may indeed predict treatment outcomes, with high expectations leading to the greatest improvement compared to moderate and low expectations. Further to this, participants’ baseline level of stress was relatively high in our study. However, future research which includes individuals with low, moderate, and severe levels of stress is necessary to determine the interplay between a diverse range of expectations and the outcome under investigation.

Information on patients’ expectations was only collected at baseline. As a result, we do not know whether expectations changed over the course of the group program. Yet, it has been proposed that patient expectations to treatment should be assessed before, during and after treatment as expectations may change over the course of treatment (Kamper et al., 2015; Laferton et al., 2017).

In addition, we did not assess whether trial participants had previously undergone hypnosis. Nevertheless, it may be important to ascertain this, as expectations may be influenced by previous exposure to hypnosis. For example, research has shown that individuals who had previously received acupuncture prior to participating in a trial investigating different briefing contents before a minimal acupuncture treatment in patients with chronic low back pain had higher expectations than those who had never received acupuncture. However, the study authors caution that higher expectations cannot be explained solely by patients’ previous experience with acupuncture, but that the relative contribution of contextual factors on patients’ pre-treatment expectations should also be considered (Zieger et al., 2022).

Although the ETS has shown to be a valid and reliable scale for measuring outcome expectations, it was originally developed in the context of acupuncture (Barth et al., 2019). While the scale has been used to determine outcome expectations across a variety of studies, there has been mixed evidence as to whether expectations predict therapeutic outcomes (de Matos et al., 2020; Barth et al., 2021; Egli et al., 2022; Zieger et al., 2022; Müller-Schrader et al., 2023). Further research should therefore be conducted using different treatment outcomes and patient populations to further explore to what extent the original scale and any modified versions are indeed able to accurately predict outcome expectations. Furthermore, the scale is not based on any theoretical models and only examines positive outcome expectations. Nonetheless, this may be problematic, as the absence of theory and negative outcome expectations could lead to important constructs being missed, thus limiting researchers’ ability to determine whether expectations do indeed predict treatment outcomes.

Lastly, we did not explore the potential influence of other variables, such as trust in the therapist. These factors may interact with expectations in complex ways that were not addressed in our research.

To our knowledge this is the first study that has explored the predictive value of expectations on hypnosis for stress reduction. It contributes to the growing understanding of the relationship between patient expectations and treatment outcomes in general, but more specifically in the field of hypnotherapy. In addition, it is based on a randomized controlled multicenter trial with high adherence rates and whose intervention was thoroughly designed and delivered by qualified hypnotherapists (physicians or psychological psychotherapists). We also recognize that the small number of study participants is a clear limitation of this secondary analysis, which may affect the generalizability of our findings. Furthermore, we did not originally plan to perform any further analysis, and therefore the results can only be interpreted in an exploratory manner.

## Conclusion

In this analysis, we found no association between participants’ expectations and perceived stress at 5 weeks in the intervention group. Our results suggest that factors contributing to the effect of hypnotherapy may have acted independently of participants’ expectations. Further research is required to explore the complex relationship between pre-therapy expectations and hypnotherapy outcomes.

## Data availability statement

The raw data supporting the conclusions of this article will be made available by the authors, without undue reservation.

## Ethics statement

The studies involving humans were approved by Ethical Approval No. EA1/067/18; Ethics Committee of the Charité – Universitätsmedizin Berlin; Charité Mitte, Charitéplatz 1 (local address: Virchowweg 10) 10117 Berlin. The studies were conducted in accordance with the local legislation and institutional requirements. The participants provided their written informed consent to participate in this study.

## Author contributions

JS: Writing – original draft, Writing – review & editing. MT: Conceptualization, Funding acquisition, Investigation, Project administration, Supervision, Writing – review & editing. BB: Conceptualization, Project administration, Supervision, Writing – review & editing. SF: Conceptualization, Funding acquisition, Investigation, Writing – review & editing. SK: Methodology, Software, Writing – original draft, Writing – review & editing.

## References

[ref1] Auer C. J. Glombiewski J. A. Doering B. K. Winkler A. Laferton J. A. C. Broadbent E.. (2016). Patients' expectations predict surgery outcomes: a Meta-analysis. Int. J. Behav. Med. 23, 49–62. doi: 10.1007/s12529-015-9500-4, PMID: 26223485

[ref2] Barth J. Kern A. Lüthi S. Witt C. M. (2019). Assessment of patients' expectations: development and validation of the expectation for treatment scale (ETS). BMJ Open 9:e026712. doi: 10.1136/bmjopen-2018-026712, PMID: 31213446 PMC6585827

[ref3] Barth J. Muff S. Kern A. Zieger A. Keiser S. Zoller M.. (2021). Effect of briefing on acupuncture treatment outcome expectations, pain, and adverse side effects among patients with chronic low Back pain: a randomized clinical trial. JAMA Netw. Open 4:e2121418. doi: 10.1001/jamanetworkopen.2021.21418, PMID: 34505889 PMC8433606

[ref4] Broughton A. (2004). Social partners sign work-related stress agreement. The European Union. Brussels

[ref5] Cohen S. Kamarck T. Mermelstein R. (1983). A global measure of perceived stress. J. Health Soc. Behav. 24, 385–396. doi: 10.2307/2136404, PMID: 6668417

[ref6] Constantino M. J. Arnkoff D. B. Glass C. R. Ametrano R. M. Smith J. A. Z. (2011). Expectations. J. Clin. Psychol. 67, 184–192. doi: 10.1002/jclp.20754, PMID: 21128304

[ref7] de Matos N. M. P. Pach D. Xing J. J. Barth J. Beyer L. E. Shi X.. (2020). Evaluating the effects of acupuncture using a dental pain model in healthy subjects - a randomized, cross-over trial. J Pain 21, 440–454. doi: 10.1016/j.jpain.2019.08.013, PMID: 31521794

[ref8] De Pascalis V. Scacchia P. Vecchio A. (2021). Influences of hypnotic suggestibility, contextual factors, and EEG alpha on placebo analgesia. Am. J. Clin. Hypn. 63, 302–328. doi: 10.1080/00029157.2020.1863182, PMID: 33999775

[ref9] Egli M. Deforth M. Keiser S. Meyenberger P. Muff S. Witt C. M.. (2022). Effectiveness of a brief hypnotic induction in third molar extraction: a randomized controlled trial (HypMol). J. Pain 23, 1071–1081. doi: 10.1016/j.jpain.2021.12.015, PMID: 35108620

[ref10] Fisch S. Binting S. Roll S. Cree M. Brinkhaus B. Teut M. (2020b). Group hypnosis for stress reduction - a feasibility study. Int. J. Clin. Exp. Hypn. 68, 493–510. doi: 10.1080/00207144.2020.1781537, PMID: 32643543

[ref11] Fisch S. Brinkhaus B. Teut M. (2017). Hypnosis in patients with perceived stress - a systematic review. BMC Complement. Altern. Med. 17:323. doi: 10.1186/s12906-017-1806-0, PMID: 28629342 PMC5477290

[ref12] Fisch S. Trivaković-Thiel S. Roll S. Keller T. Binting S. Cree M.. (2020a). Group hypnosis for stress reduction and improved stress coping: a multicenter randomized controlled trial. BMC Complement Med. Ther. 20:344. doi: 10.1186/s12906-020-03129-6, PMID: 33187503 PMC7664040

[ref13] Frisaldi E. Piedimonte A. Benedetti F. (2015). Placebo and nocebo effects: a complex interplay between psychological factors and neurochemical networks. Am. J. Clin. Hypn. 57, 267–284. doi: 10.1080/00029157.2014.976785, PMID: 25928679

[ref14] Gnall K. E. Sacco S. J. Park C. L. Mazure C. M. Hoff R. A. (2023). Life meaning and mental health in post-9/11 veterans: the mediating role of perceived stress. Anxiety Stress Coping 36, 743–756. doi: 10.1080/10615806.2022.2154341, PMID: 36542555

[ref15] Kamper S. J. Kongsted A. Haanstra T. M. Hestbaek L. (2015). Do recovery expectations change over time? Eur. Spine J. 24, 218–226. doi: 10.1007/s00586-014-3380-1, PMID: 24913213

[ref16] Kirsch I. (1985). Response expectancy as a determinant of experience and behavior. Am. Psychol. 40, 1189–1202. doi: 10.1037/0003-066X.40.11.1189, PMID: 38584918

[ref17] Koban L. Jepma M. Geuter S. Wager T. D. (2017). What's in a word? How instructions, suggestions, and social information change pain and emotion. Neurosci. Biobehav. Rev. 81, 29–42. doi: 10.1016/j.neubiorev.2017.02.014, PMID: 29173508 PMC5706563

[ref18] Laferton J. A. Kube T. Salzmann S. Auer C. J. Shedden-Mora M. C. (2017). Patients' expectations regarding medical treatment: a critical review of concepts and their assessment. Front. Psychol. 8:233. doi: 10.3389/fpsyg.2017.00233, PMID: 28270786 PMC5318458

[ref19] Mazure C. M. Husky M. M. Pietrzak R. H. (2023). Stress as a risk factor for mental disorders in a gendered environment. JAMA Psychiatry 80, 1087–1088. doi: 10.1001/jamapsychiatry.2023.3138, PMID: 37672277

[ref20] Mondloch M. V. Cole D. C. Frank J. W. (2001). Does how you do depend on how you think you'll do? A systematic review of the evidence for a relation between patients' recovery expectations and health outcomes. CMAJ 165, 174–179. PMID: 11501456 PMC81284

[ref21] Müller-Schrader M. Heinzle J. Müller A. Lanz C. Häussler O. Sutter M.. (2023). Individual treatment expectations predict clinical outcome after lumbar injections against low back pain. Pain 164, 132–141. doi: 10.1097/j.pain.0000000000002674, PMID: 35543638

[ref22] Olendzki N. Elkins G. R. Slonena E. Hung J. Rhodes J. R. (2020). Mindful hypnotherapy to reduce stress and increase mindfulness: a randomized controlled pilot study. Int. J. Clin. Exp. Hypn. 68, 151–166. doi: 10.1080/00207144.2020.1722028, PMID: 32223617

[ref23] Payrau B. Quere N. Breton E. Payrau C. (2017). Fasciatherapy and reflexology compared to hypnosis and music therapy in daily stress management. Int. J. Ther. Massage Bodywork 10, 4–13. doi: 10.3822/ijtmb.v10i3.368, PMID: 28912904 PMC5593310

[ref24] Petrovic P. Dietrich T. Fransson P. Andersson J. Carlsson K. Ingvar M. (2005). Placebo in emotional processing— induced expectations of anxiety relief activate a generalized modulatory network. Neuron 46, 957–969. doi: 10.1016/j.neuron.2005.05.023, PMID: 15953423

[ref25] Popescu C. A. Tegzeșiu A. M. Suciu S. M. Covaliu B. F. Armean S. M. Uță T. A.. (2023). Evolving mental health dynamics among medical students amid COVID-19: A comparative analysis of stress, depression, and alcohol use among medical students. Medicina (Kaunas) 59:1854. doi: 10.3390/medicina59101854 37893572 PMC10608214

[ref26] Sara J. D. S. Lerman L. O. Lerman A. (2023). What can biologic aging tell us about the effects of mental stress on vascular health. Hypertension 80, 2515–2522. doi: 10.1161/HYPERTENSIONAHA.123.19418, PMID: 37814855

[ref27] Sliwinski J. R. Elkins G. R. (2017). Hypnotherapy to reduce hot flashes: examination of response expectancies as a mediator of outcomes. J. Evid. Based Complementary Altern. Med. 22, 652–659. doi: 10.1177/2156587217708523, PMID: 28528570 PMC5871284

[ref28] Slonena E. E. Elkins G. R. (2021). Effects of a brief mindful hypnosis intervention on stress reactivity: a randomized active control study. Int. J. Clin. Exp. Hypn. 69, 453–467. doi: 10.1080/00207144.2021.1952845, PMID: 34330204

[ref29] Tracey I. (2010). Getting the pain you expect: mechanisms of placebo, nocebo and reappraisal effects in humans. Nat. Med. 16, 1277–1283. doi: 10.1038/nm.2229, PMID: 20948533

[ref30] Vahdat S. Fathi M. Feyzi Z. Shakeri M. T. Tafazoli M. (2022). The effect of hypnosis on perceived stress in women with preeclampsia. J. Educ. Health Promot. 11:111. doi: 10.4103/jehp.jehp_744_20, PMID: 35573632 PMC9093662

[ref31] Wager T. D. Scott D. J. Zubieta J. K. (2007). Placebo effects on human mu-opioid activity during pain. Proc. Natl. Acad. Sci. USA 104, 11056–11061. doi: 10.1073/pnas.0702413104, PMID: 17578917 PMC1894566

[ref32] Wagner-Link A. (2017). Stress Belastungen besser bewältigen: Techniker Krankenkasse. Hamburg

[ref33] Walther L. M. Wirtz P. H. (2023). Physiological reactivity to acute mental stress in essential hypertension-a systematic review. Front. Cardiovasc. Med. 10:1215710. doi: 10.3389/fcvm.2023.1215710, PMID: 37636310 PMC10450926

[ref34] Wohlers K. Hombrecher M. (2016). Entspann dich. Deutschland TK-Stressstudie. Techniker Krankenkasse. Hamburg

[ref35] Zieger A. Kern A. Barth J. Witt C. M. (2022). Do patients' pre-treatment expectations about acupuncture effectiveness predict treatment outcome in patients with chronic low back pain? A secondary analysis of data from a randomised controlled clinical trial. PLoS One 17:e0268646. doi: 10.1371/journal.pone.0268646, PMID: 35594274 PMC9122231

